# Supplementary material for: SAHA increases chaperone expression and reduces Z-alpha-1-antitrypsin polymers in a patient specific iPSC-based liver model for alpha-1-antitrypsin deficiency
Source: Stem Cell Res Ther. 2026 Oct 27;17:386. doi: 10.1186/s13287-026-05365-y (PMC13718072; doi:10.1186/s13287-026-05365-y)
Supplement: Supplementary file 1 [file 13287_2026_5365_MOESM1_ESM.pdf]

Suppl. Fig. S1A

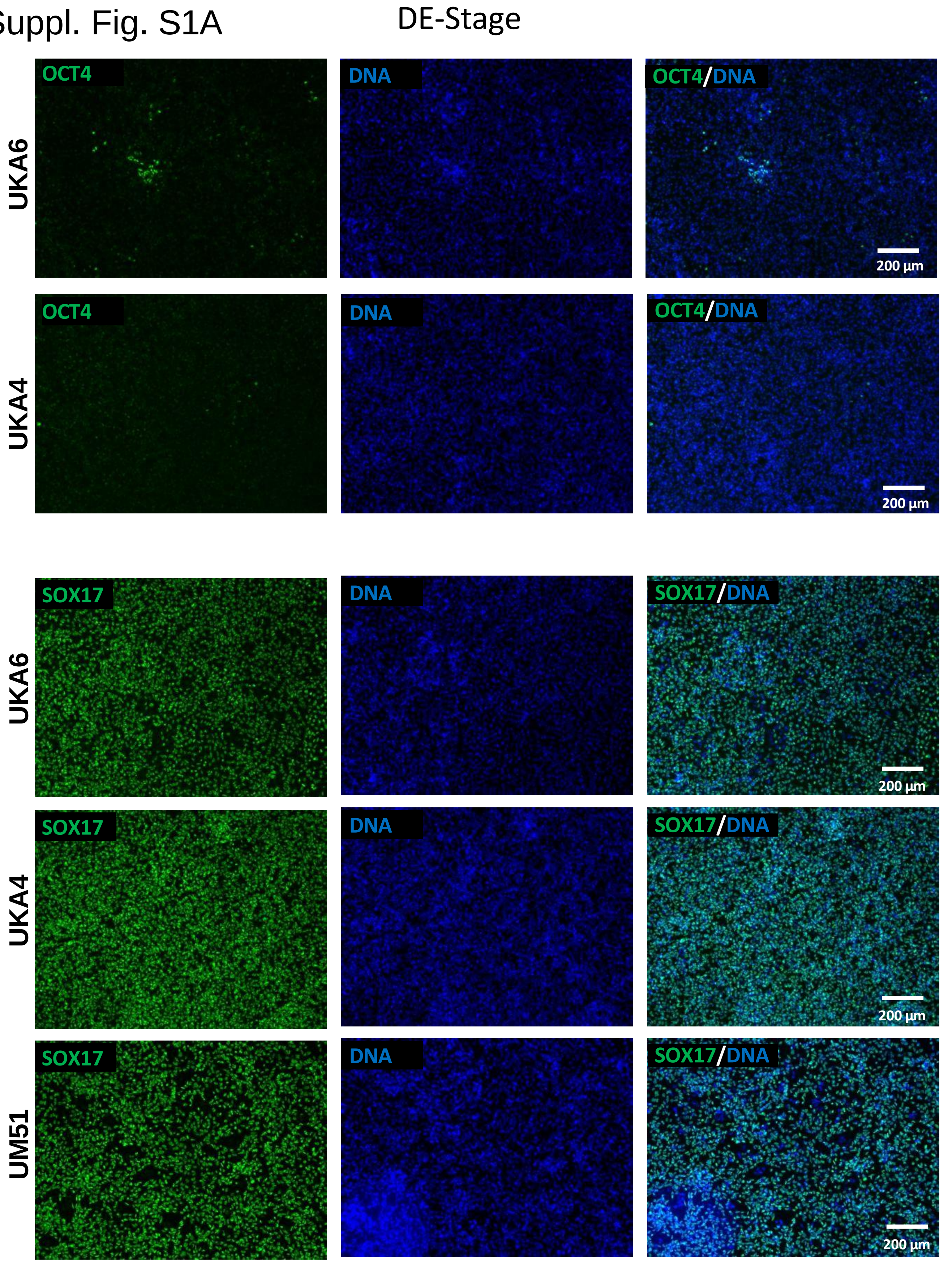

Suppl. Fig. S1B

HE-Stage

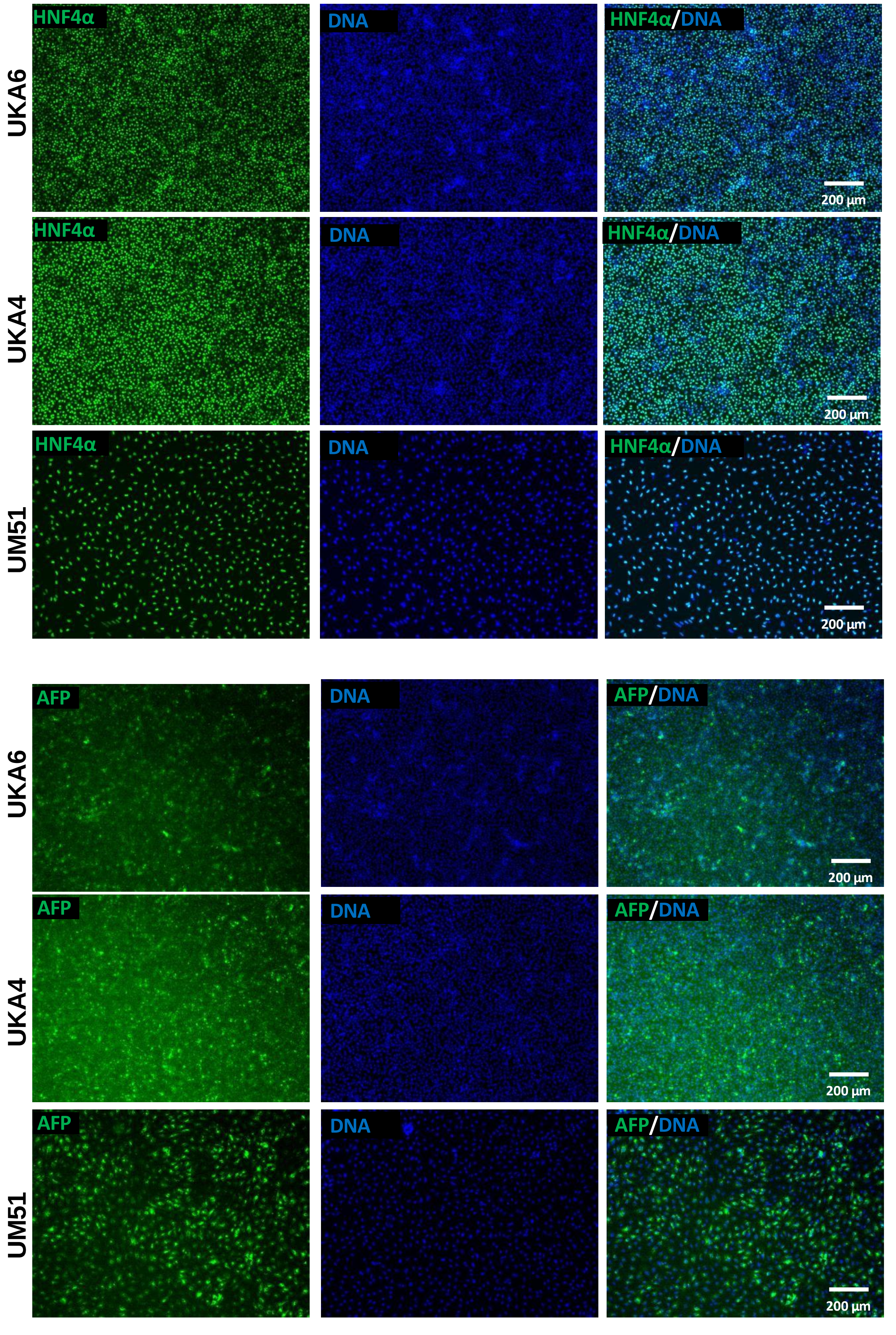

Suppl. Fig. S1C

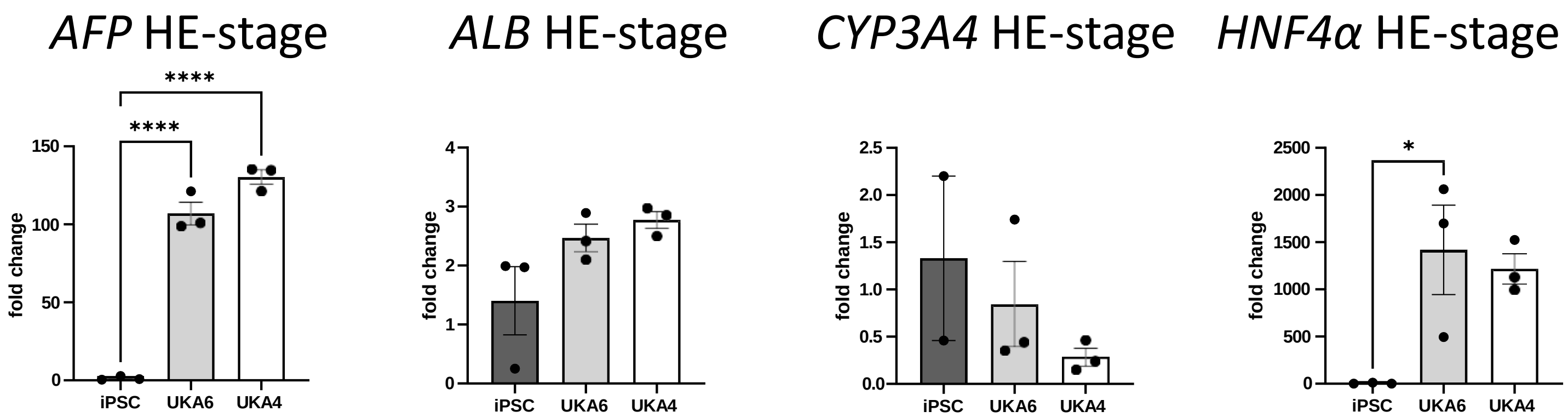

Suppl. Fig. S1D

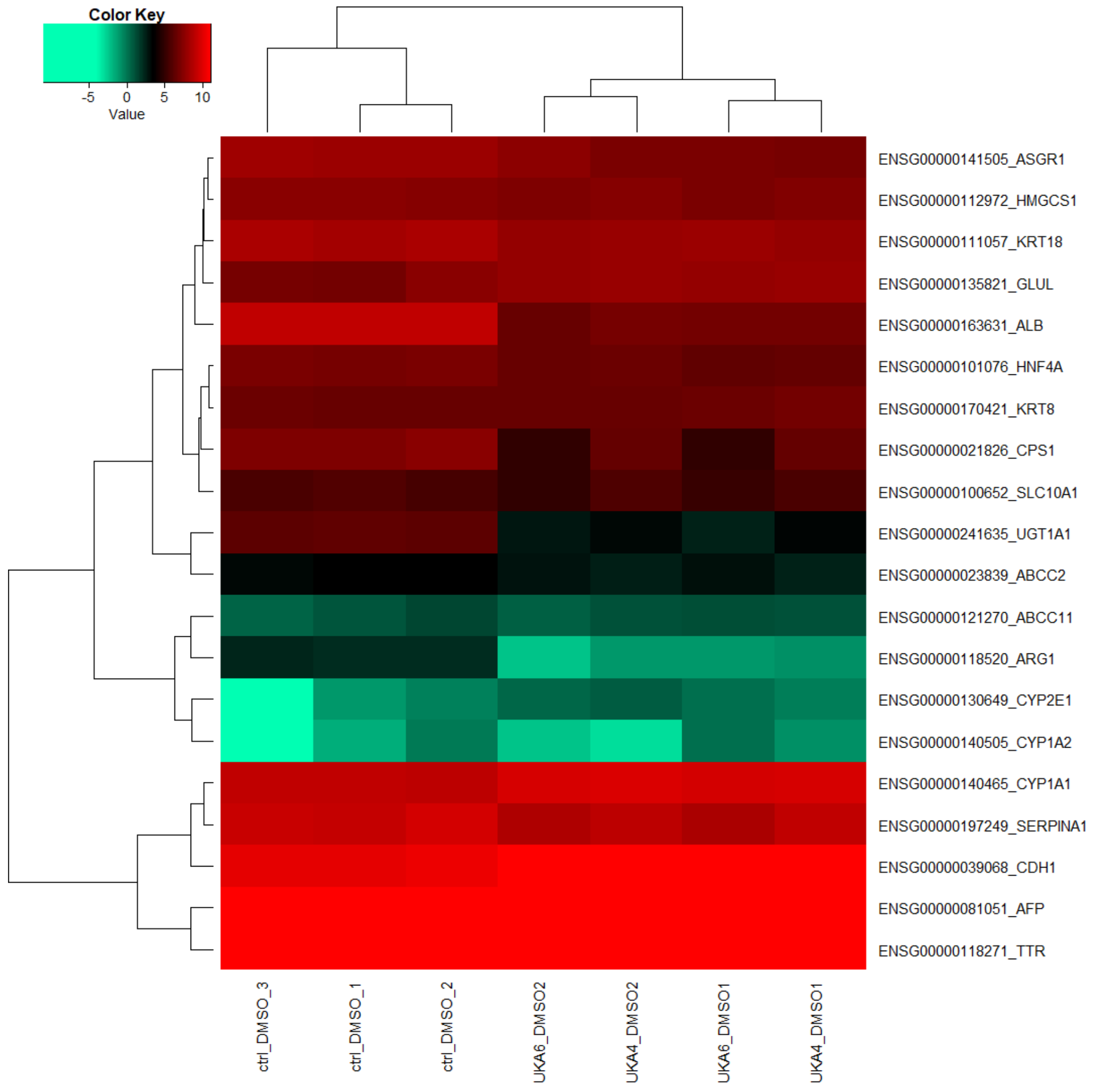

Suppl. Fig. S1: Detailed comparison of HLC differentiation. (A) At DE-stage, cells have mostly lost the pluripotency marker Octamer binding transcription factor 4 (OCT4) and express SRY-box transcription factor 17 (SOX17). **(B)** In HE stage the cells are positive for Hepatocyte nuclear factor 4 alpha (HNF4α) and alpha-fetoprotein (AFP). Scale bar = 200 µm. **(C)** Relative mRNA expression of AFP, ALB, HNF4a, and CYP3A4 in HE-stage, normalized to the housekeeping gene *RPLP0*. Bar plots show mean of triplicates for each donor ±SEM. One-way ANOVA was performed to calculate significances (*p < 0.05, ****p < 0.0001). **(D)** Heatmap demonstrating expression of various characteristic genes for hepatocytes in all 3 cell lines in HLC-stage.

Suppl. Fig. S2

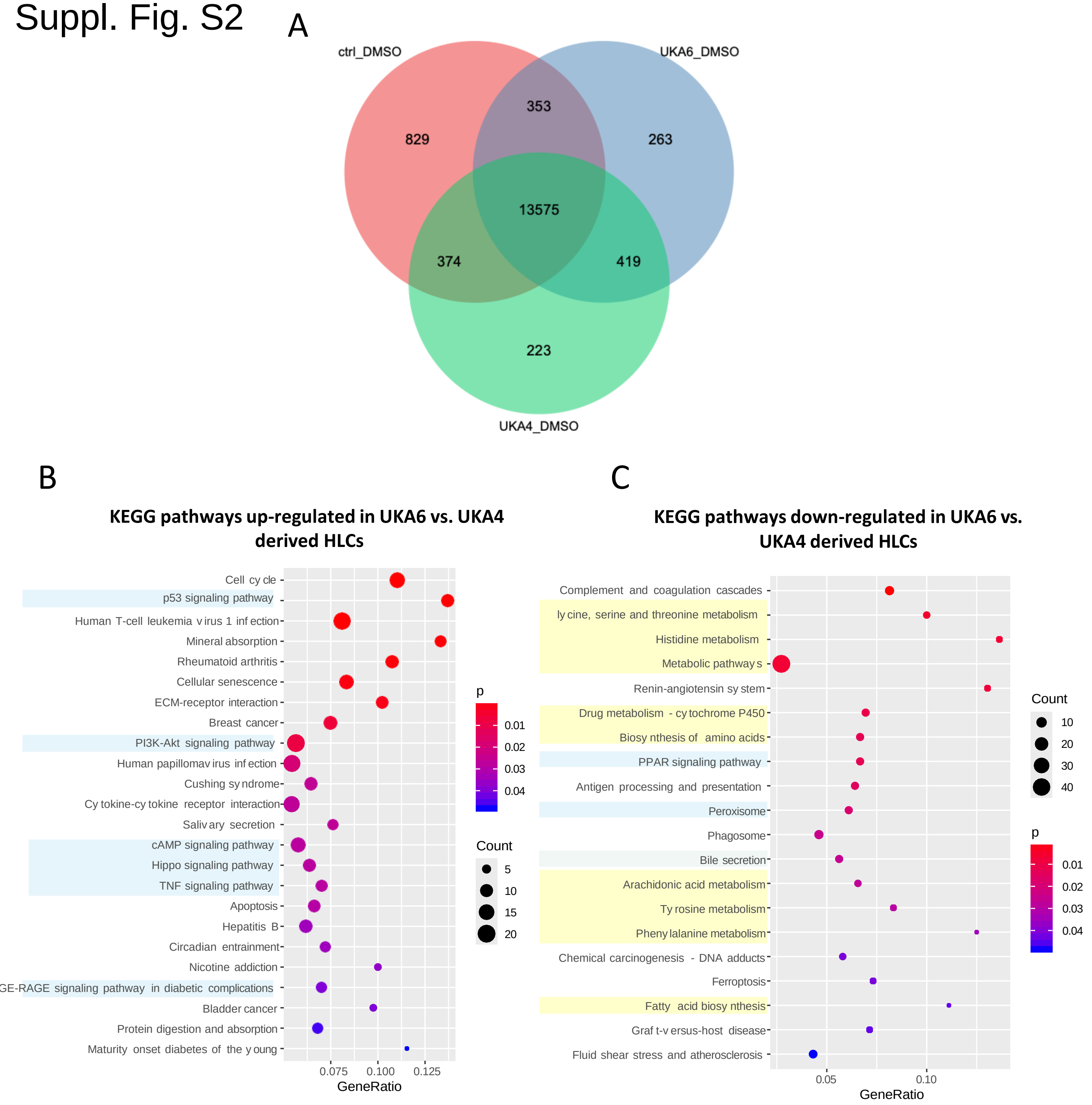

Suppl. Fig. S2: Differences in gene expression between the two AATD-patient derived HLCs. (A) Venn diagram indicating number of genes exclusively expressed in control (829), UKA6 (263), or UKA 4 (223) HLCs **(B)** KEGG pathways significantly up-regulated in UKA6-derived HLCs. **(C)** KEGG pathways significantly down-regulated in UKA6-derived HLCs. Pathways associated with signalling are highlighted in blue, with metabolism in yellow, with (bile) secretion in green. For full data set, please refer to Suppl. Table S5.

Suppl. Fig. S3

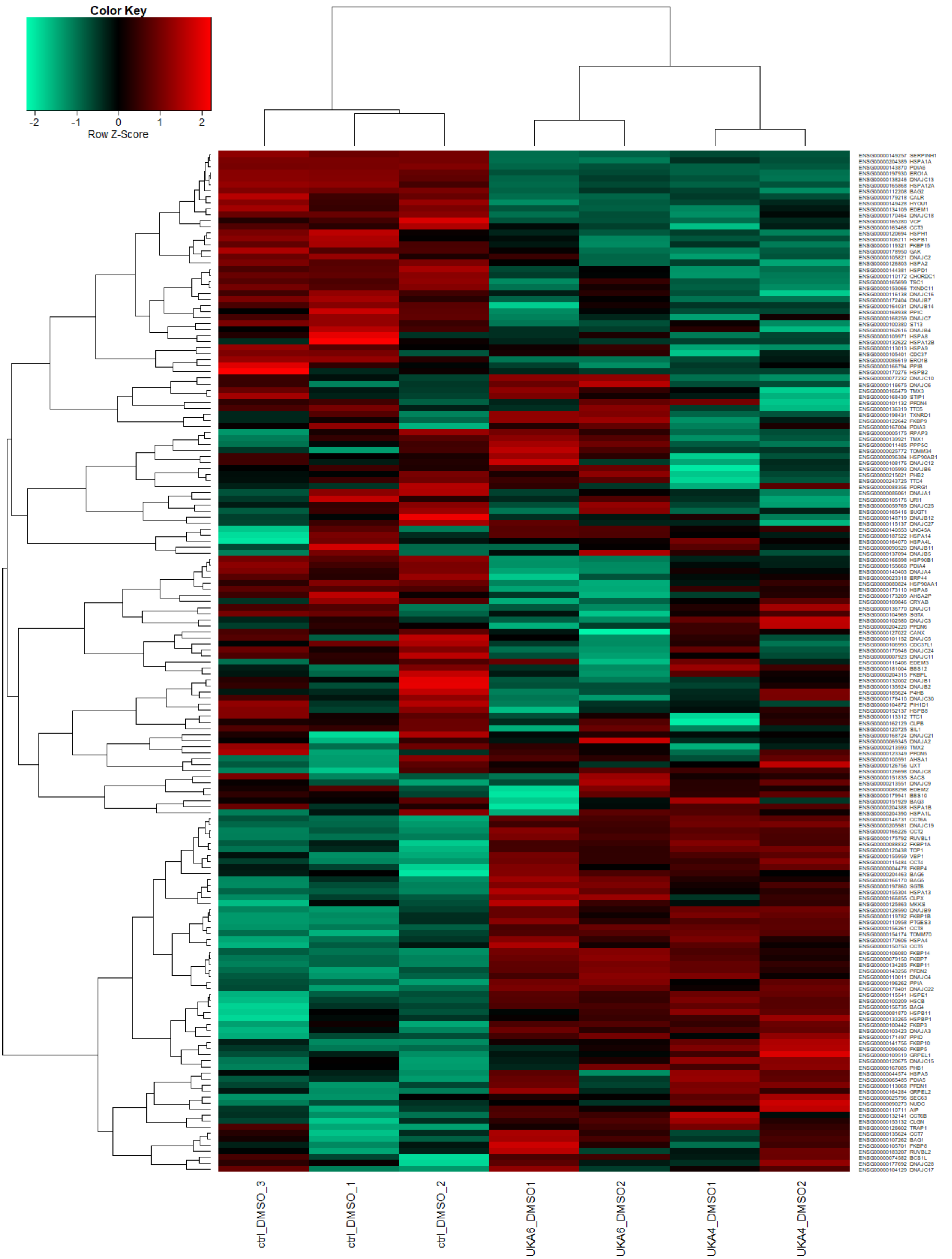

Suppl. Fig. S3: Heatmap of heat-shock protein genes shows clear clustering according to AAT genotype

Suppl. Fig. S4

A **STRING network of genes common in at least 2 of the transcriptomic pathways down-regulated in AATD-patient derived HLCs**

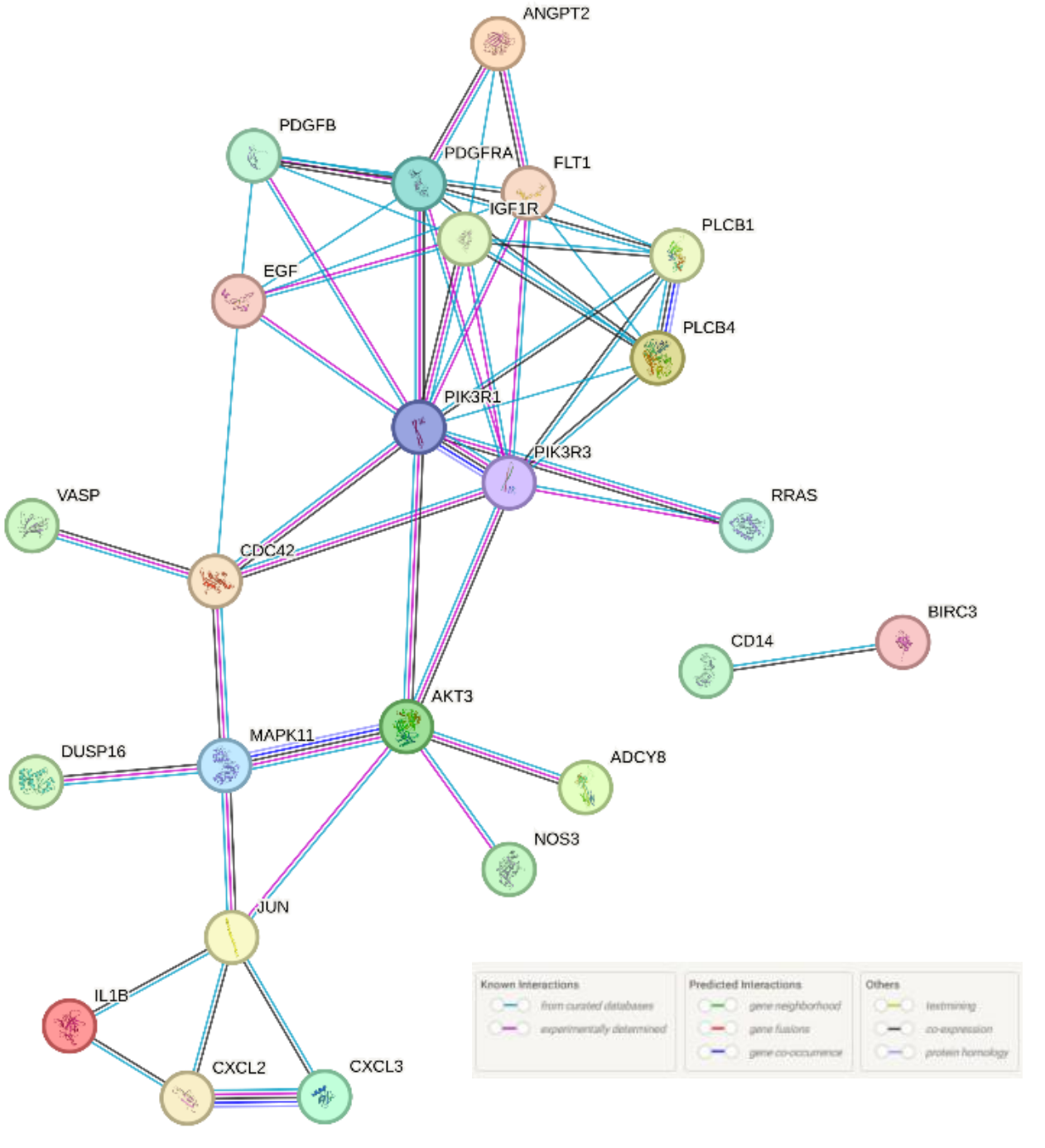

Suppl. Fig. S4: STRING network of genes common in at least 2 of the transcriptomic pathways down-regulated in AATD patient-derived HLCs

Suppl Fig. S5 UKA 6

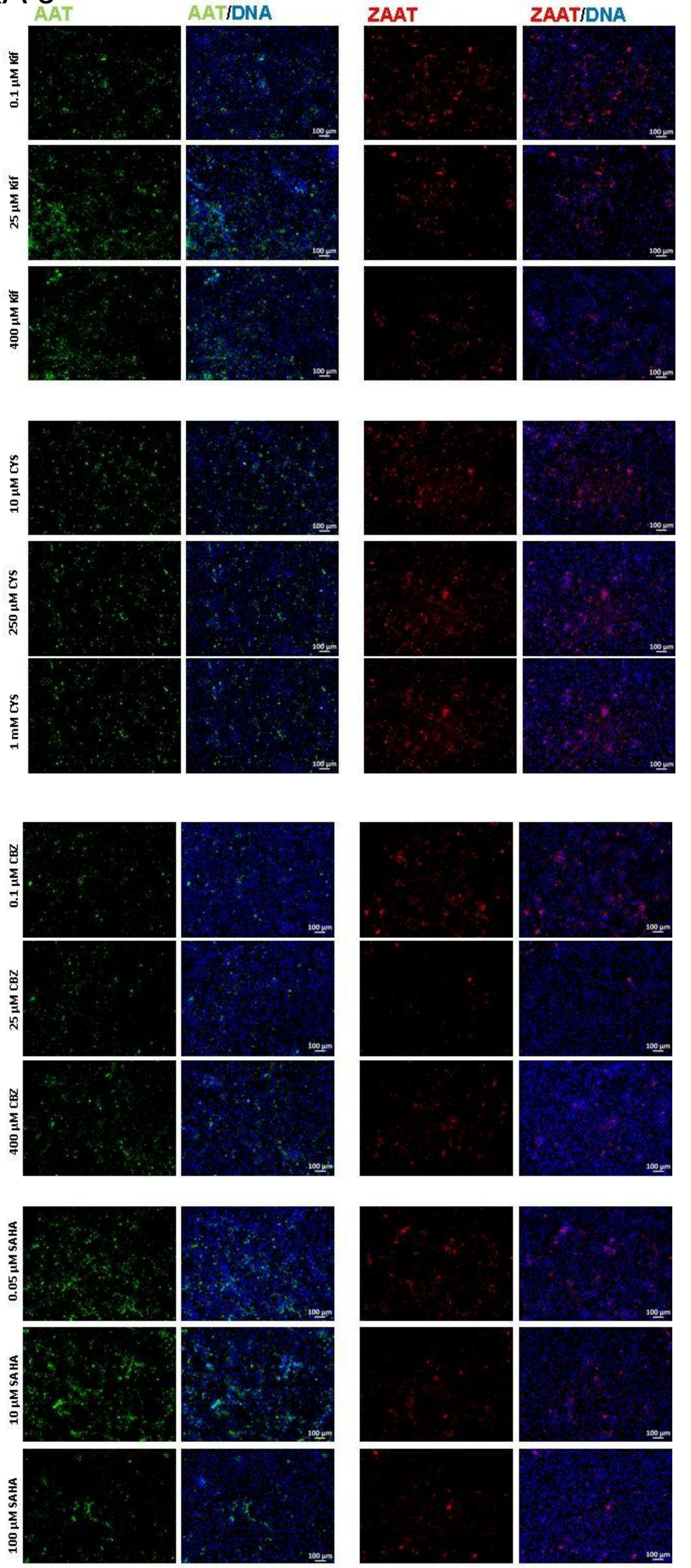

Suppl. Fig. S5: Concentration testing of the 4 compounds. ICC was performed to test changes in the amount of ZAAT (red) and AAT (green) after treatment with the indicated small molecules in relation to their respective controls (SAHA, CBZ: ctrl = DMSO, Kif: ctrl = H_2O, CYS: ctrl = HCl). For each compound in total 9 different concentrations ranging between 0.1 µM – 400 µM for CBZ and KIF, 0.05 µM – 100 µM for SAHA, and 10 µM – 1 mM for CYS were tested on UKA6 HLCs. Depicted are the lowest and highest concentrations as well as the concentration selected for the experiments.

Suppl. Fig. S6 UKA4

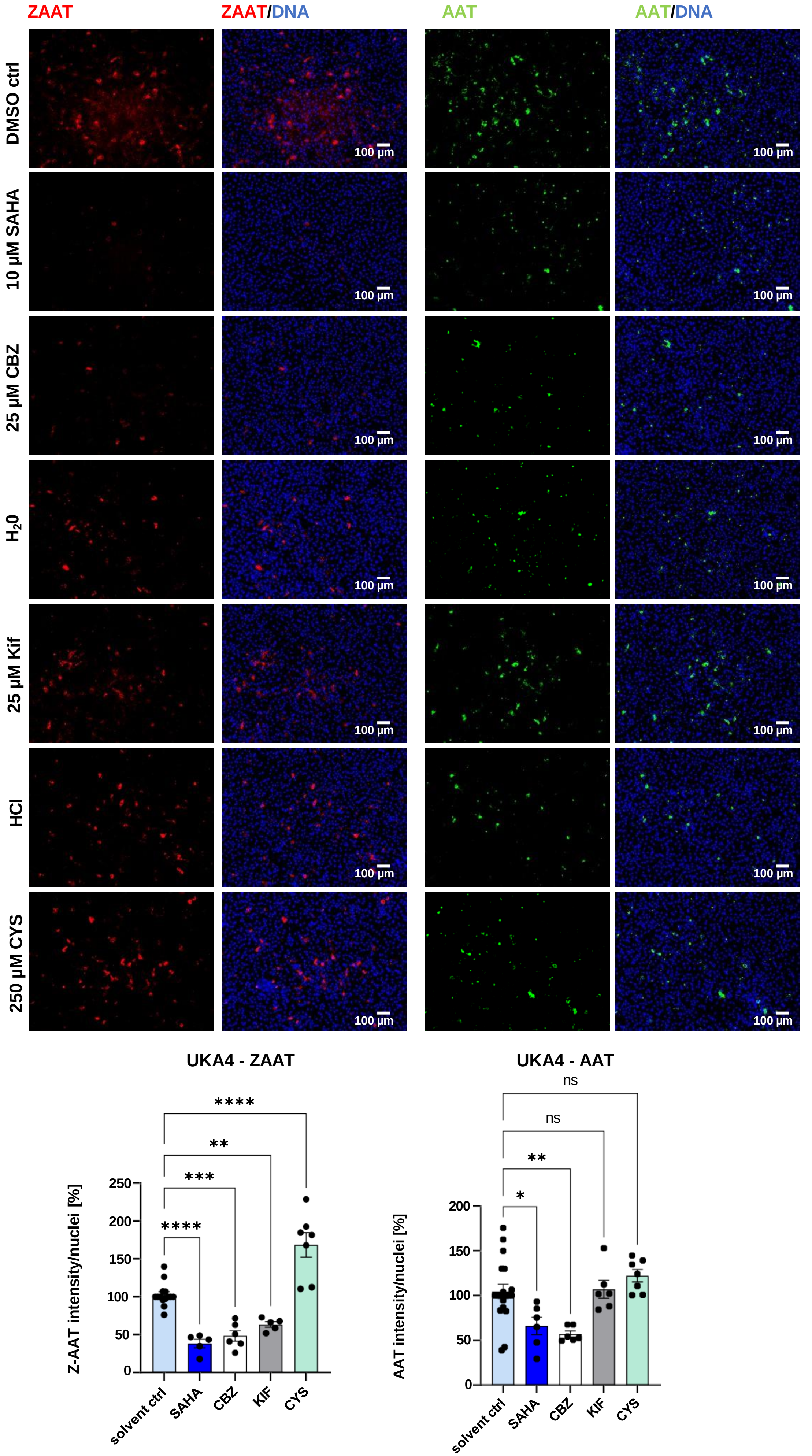

Suppl. Fig. S6: Small molecule treatment influences the amount of ZAAT and AAT in patient-derived HLCs. (A) ICC reveals changes in the amount of ZAAT (red) and AAT (green) after treatment with the indicated small molecules in relation to their respective controls (SAHA, CBZ: ctrl = DMSO, Kif: ctrl = H_2O, CYS: ctrl = HCl). **(B)** Quantification of ZAAT and AAT, N= 5-19, bars represent mean ±SEM. Two-way ANOVA was performed to calculate significances (*$p < 0.05$, **$p < 0.01$, ***$p < 0.001$, ****$p < 0.0001$).

Suppl. Fig. S7

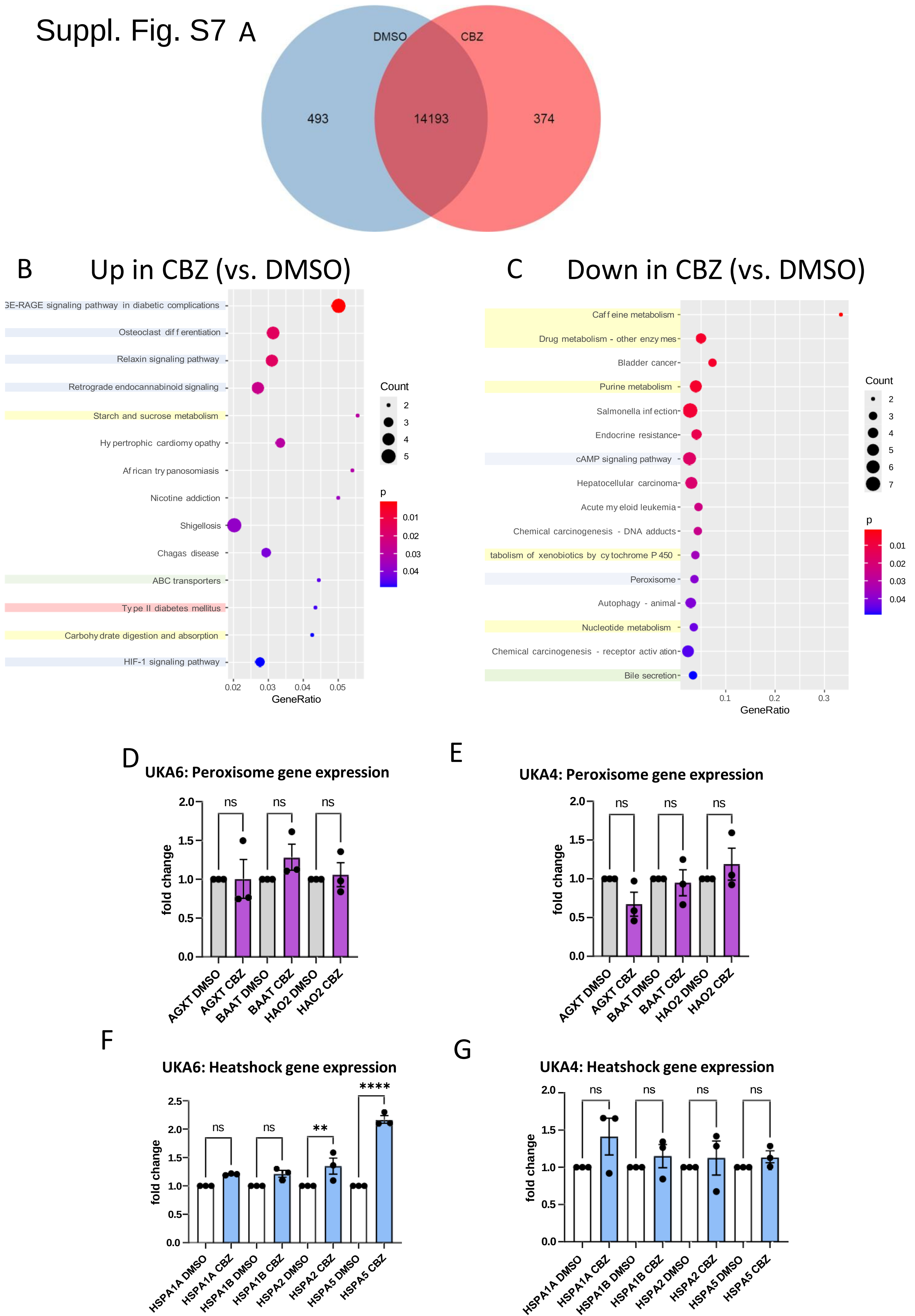
A
DMSO
CBZ
493
14193
374
B
Up in CBZ (vs. DMSO)
Osteoclast differentiation
Relaxin signaling pathway
Retrograde endocannabinoid signaling
Starch and sucrose metabolism
Hypertrophic cardiomyopathy
African trypanosomiasis
Nicotine addiction
Shigellosis
Chagas disease
ABC transporters
Type II diabetes mellitus
Carbohydrate digestion and absorption
HIF-1 signaling pathway
0.02
0.03
0.04
0.05
GeneRatio
Count
2
3
4
5
p
0.01
0.02
0.03
0.04
C
Down in CBZ (vs. DMSO)
Caffeine metabolism
Drug metabolism - other enzymes
Bladder cancer
Purine metabolism
Salmonella infection
Endocrine resistance
cAMP signaling pathway
Hepatocellular carcinoma
Acute myeloid leukemia
Chemical carcinogenesis - DNA adducts
Peroxisome
Autophagy - animal
Nucleotide metabolism
Chemical carcinogenesis - receptor activation
Bile secretion
0.1
0.2
0.3
GeneRatio
Count
2
3
4
5
6
7
p
0.01
0.02
0.03
0.04
D
UKA6: Peroxisome gene expression
fold change
ns
AGXT DMSO
AGXT CBZ
BAAT DMSO
BAAT CBZ
HAO2 DMSO
HAO2 CBZ
E
UKA4: Peroxisome gene expression
F
UKA6: Heatshock gene expression
**
****
HSPA1A DMSO
HSPA1A CBZ
HSPA1B DMSO
HSPA1B CBZ
HSPA2 DMSO
HSPA2 CBZ
HSPA5 DMSO
HSPA5 CBZ
G
UKA4: Heatshock gene expression

Suppl. Fig. S7: CBZ treatment increases the expression of genes associated with signalling pathways and reduces metabolism associated genes. (A) Venn diagram indicating number of genes exclusively expressed in HLCs after CBZ treatment (347), uniquely expressed in control conditions (493) and commonly expressed (14,193) **(B)** KEGG pathways significantly up-regulated after CBZ treatment. **(C)** KEGG pathways significantly down-regulated after CBZ treatment. Pathways associated with signalling are highlighted in blue, with metabolism in yellow, with (bile) secretion in green, and with diabetes in red. For full data set, please refer to Suppl. Table S6. **(D-G)** Relative mRNA expression of heat shock protein genes (D,E) and peroxisome pathway genes (F,G) normalized to the housekeeping gene *RPLP0*. Bar plots show mean of triplicates ±SEM. Two-way ANOVA was performed to calculate significances. Only those between treated/untreated samples are shown (**$p < 0.01$, ****$p < 0.0001$).

Suppl. Fig. S8

A

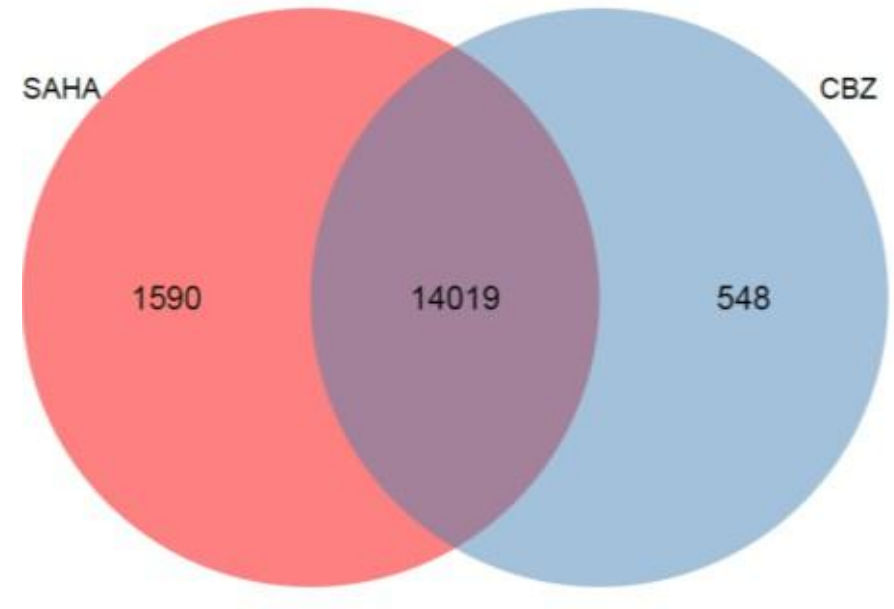

B

KEGG pathways up-regulated in SAHA- vs. CBZ-treated HLCs

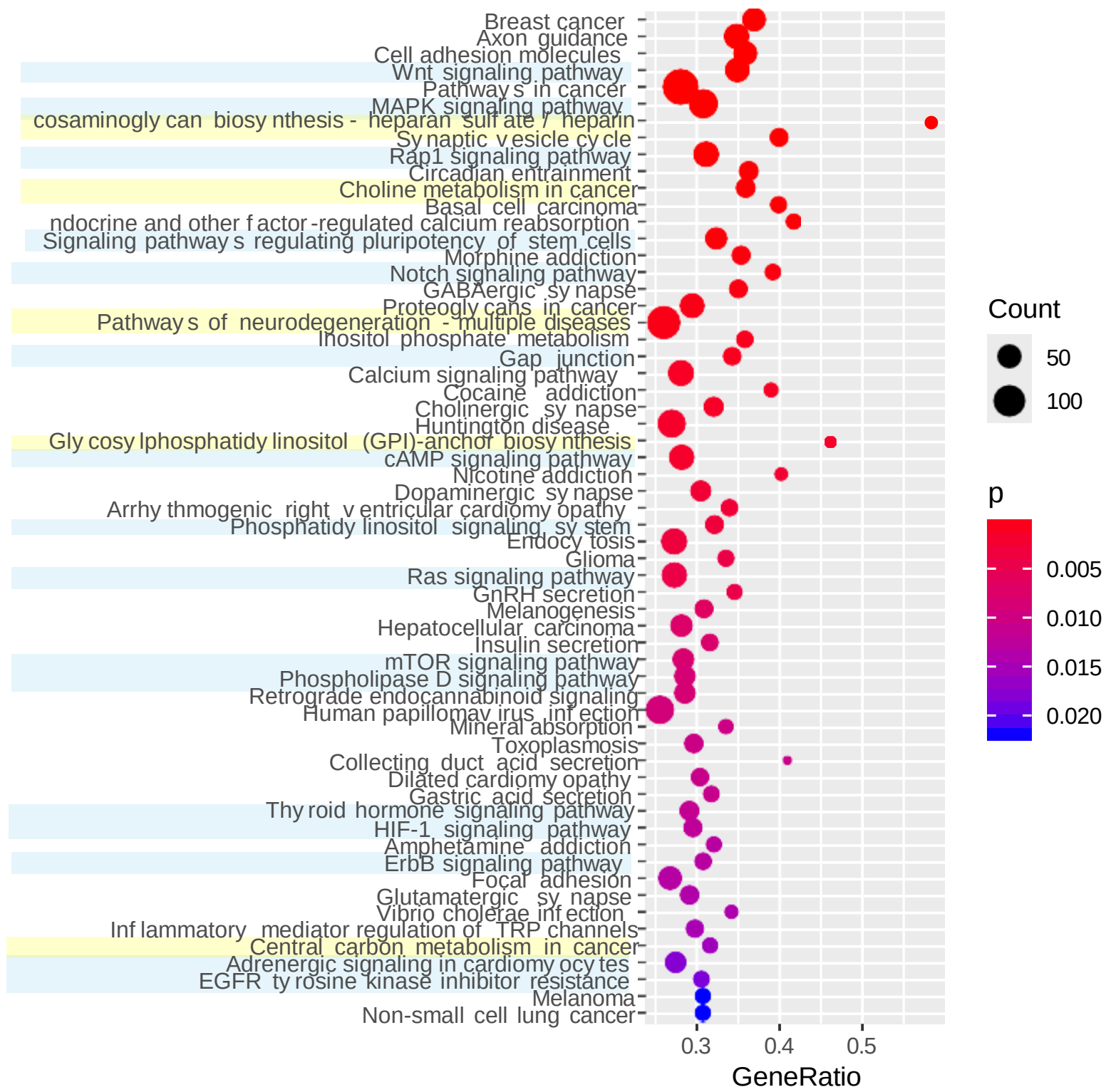

C

KEGG pathways down-regulated in SAHA- vs. CBZ-treated HLCs

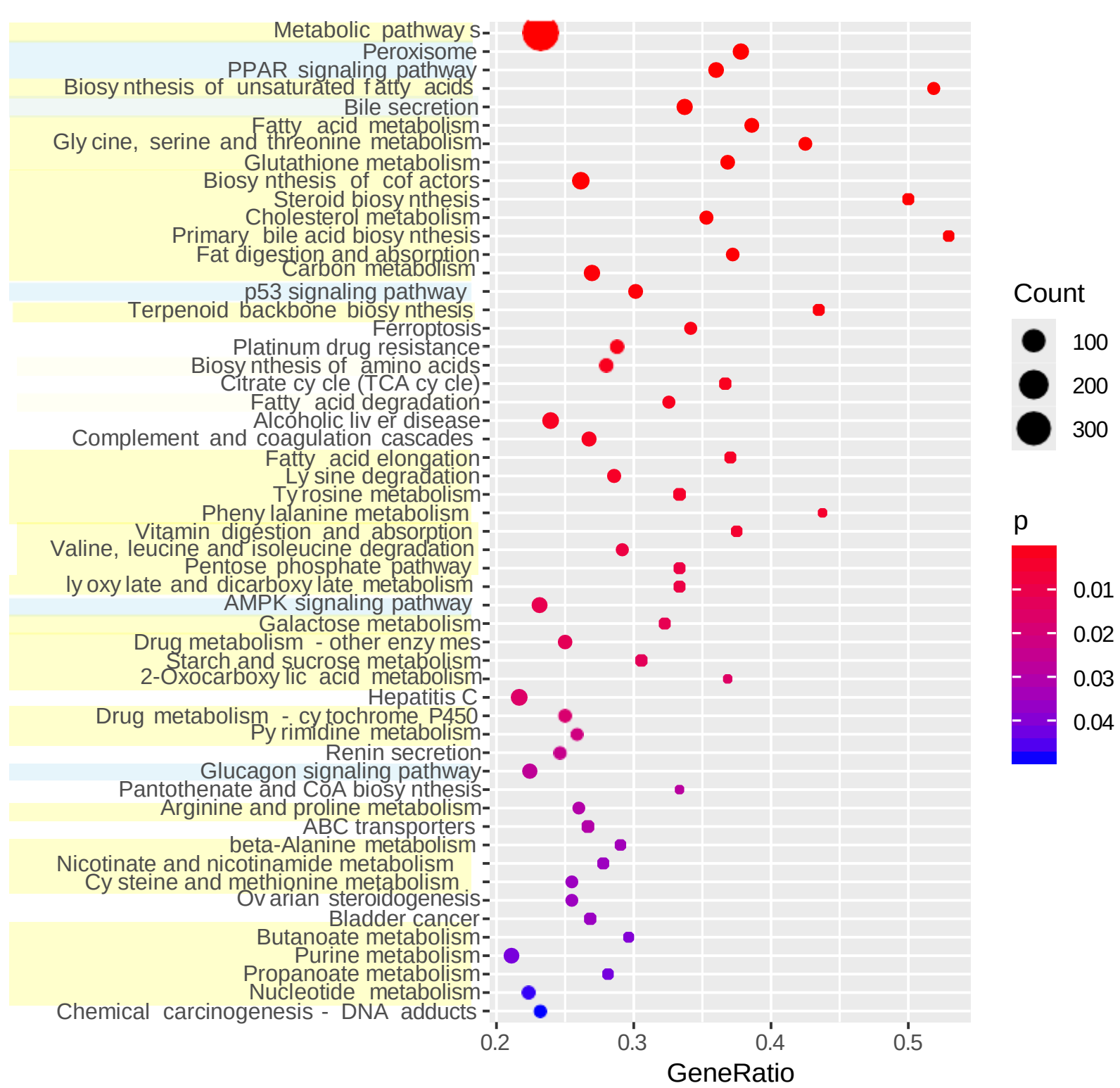

Suppl. Fig. S8: Comparison of the effects of SAHA- and CBZ-treatment on AATD-patient derived HLCs. **(A)** Venn diagram indicating number of genes exclusively expressed in HLCs after SAHA- (1,590) or CBZ (548) treatment and commonly expressed (14,019) **(B)** KEGG pathways significantly up-regulated after SAHA vs. CBZ treatment. **(C)** KEGG pathways significantly down-regulated after SAHA vs. CBZ treatment. Pathways associated with signalling are highlighted in blue, with metabolism in yellow, and with (bile) secretion in green. For full data set, please refer to Suppl. Table S6.

Suppl. Fig. S9

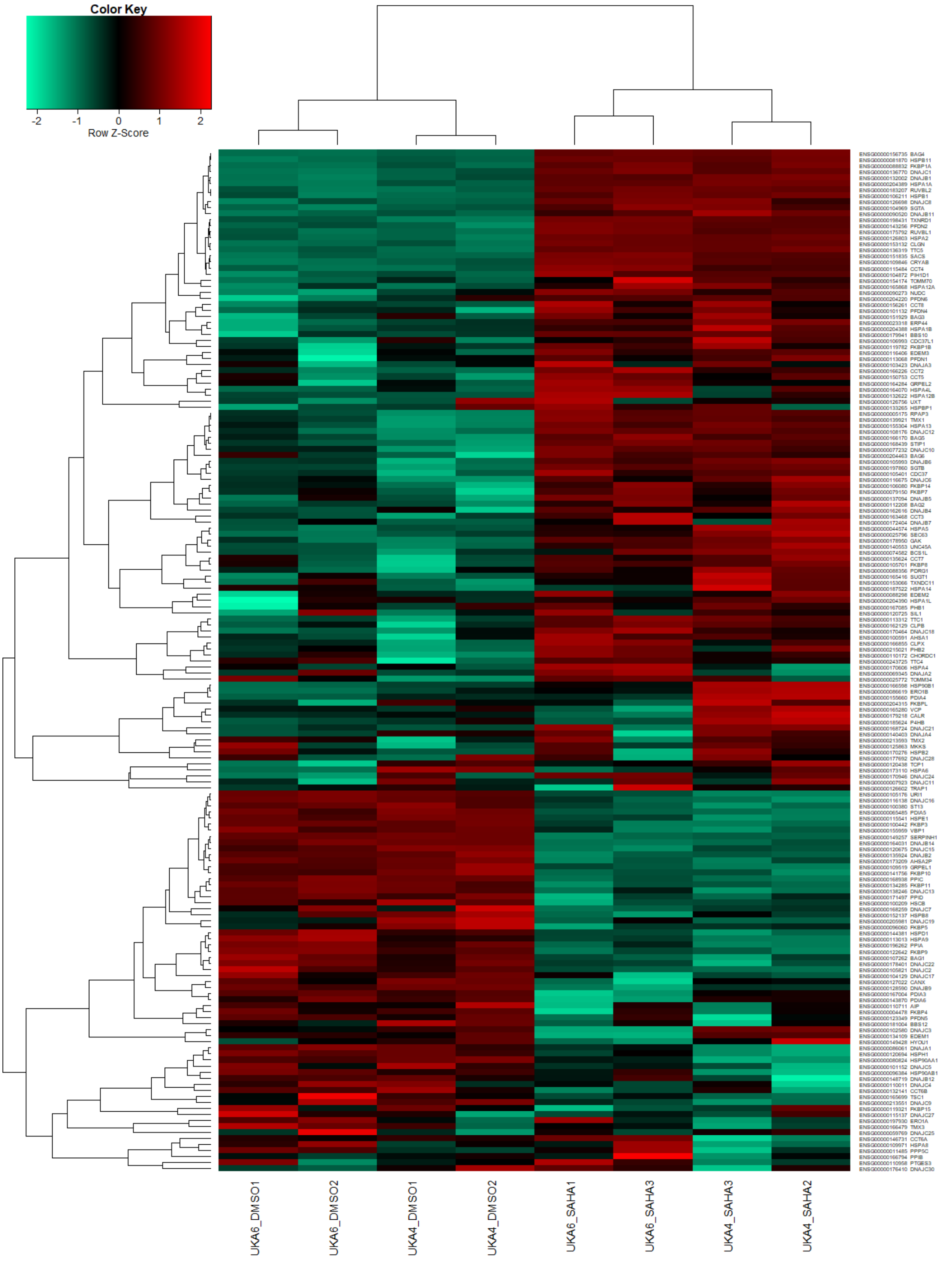

Suppl. Fig. S9: Heatmap of heat-shock protein genes shows clear clustering according to SAHA vs DMSO treatment

Suppl. Fig. S10

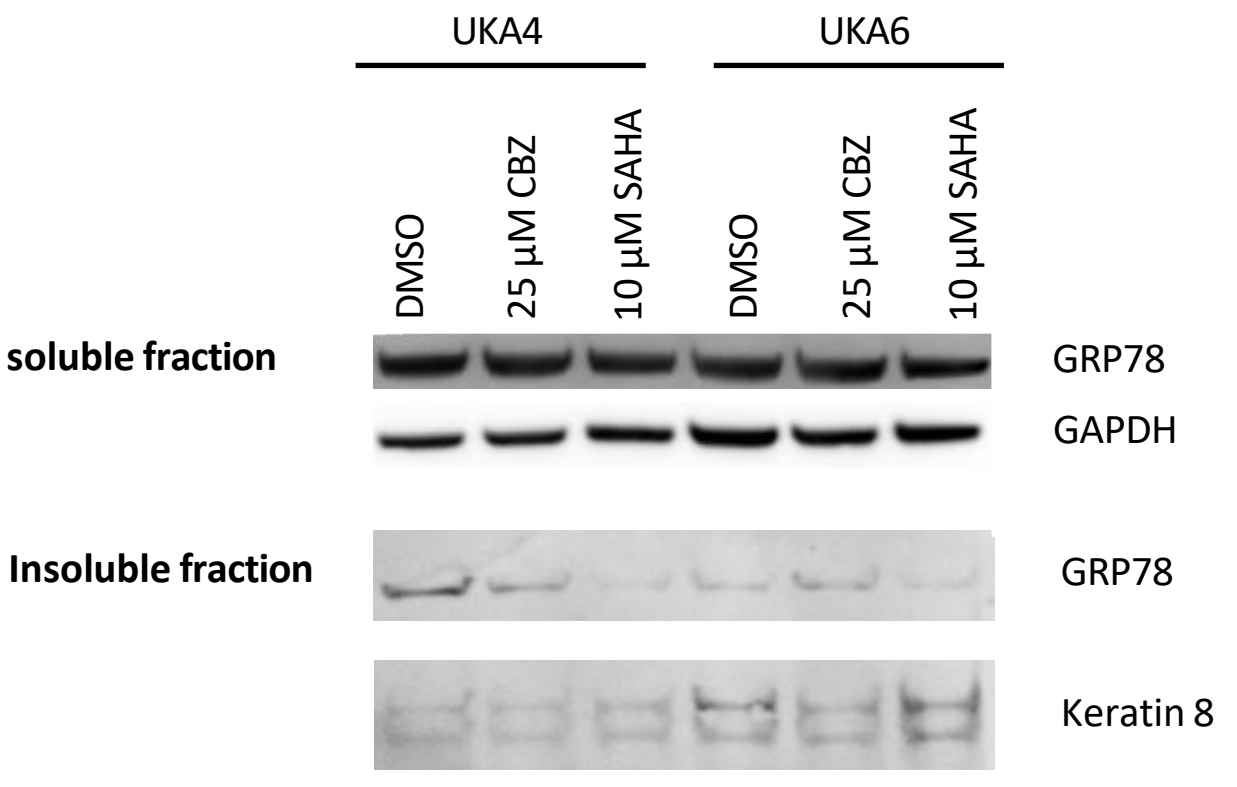

Suppl. Fig. S10: Western blot for the Triton X soluble (up) and insoluble (down) fraction of patient-derived HLCs indicate similar levels of GRP78 after CBZ or SAHA treatment. GAPDH and keratin 8 served as respective housekeeping genes. n=1 (pool of 2 independent wells). Uncropped blot is shown in Suppl. Fig. S13.

Suppl. Fig. S11

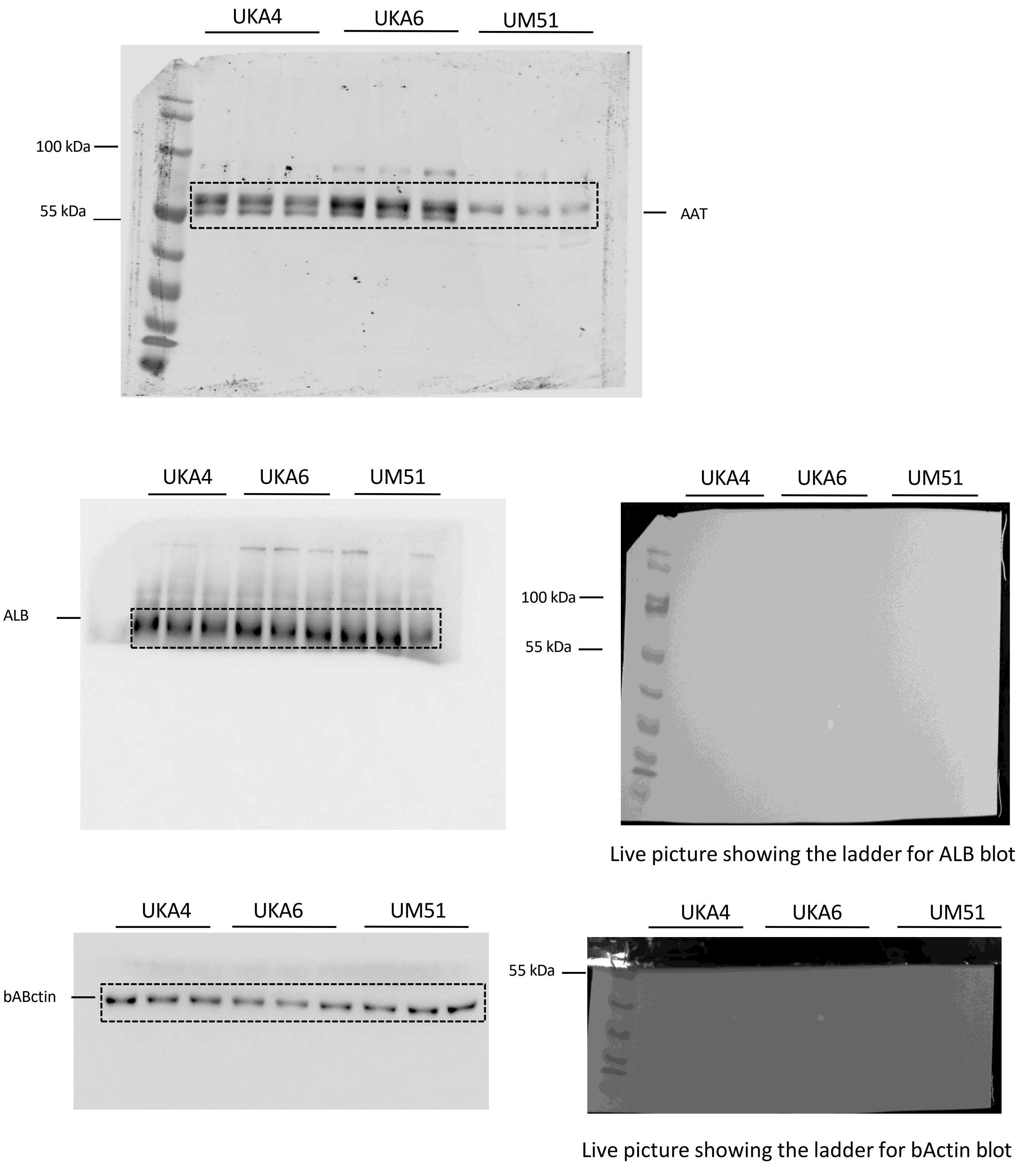

Suppl. Fig. S11: Uncropped Western blots from Fig. 2B. Dashed lines indicate cropped regions.

Suppl. Fig. S12

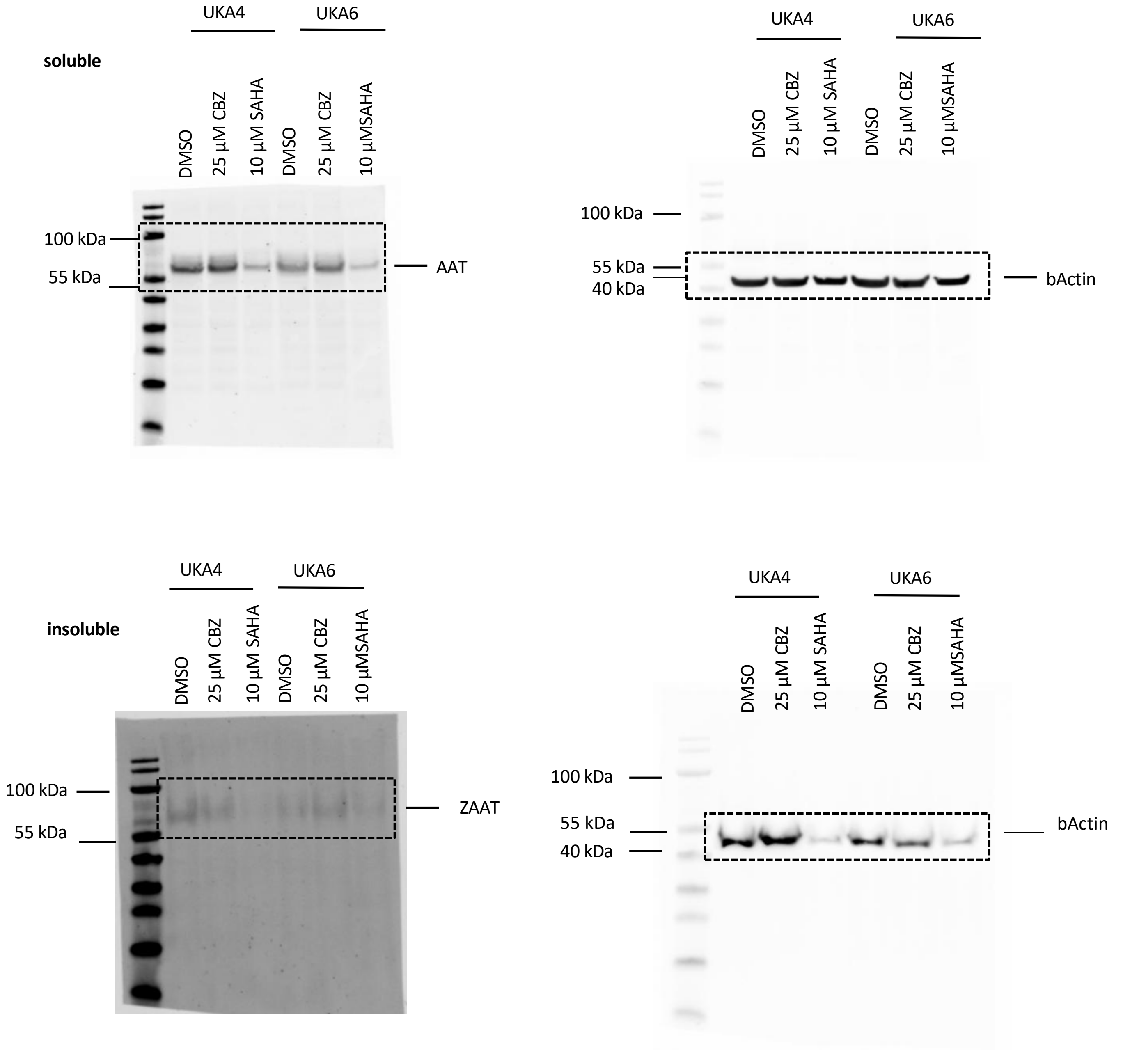

Suppl. Fig. S12: Uncropped Western blots from Fig. 4D. Dashed lines indicate cropped regions.

Suppl. Fig. S13

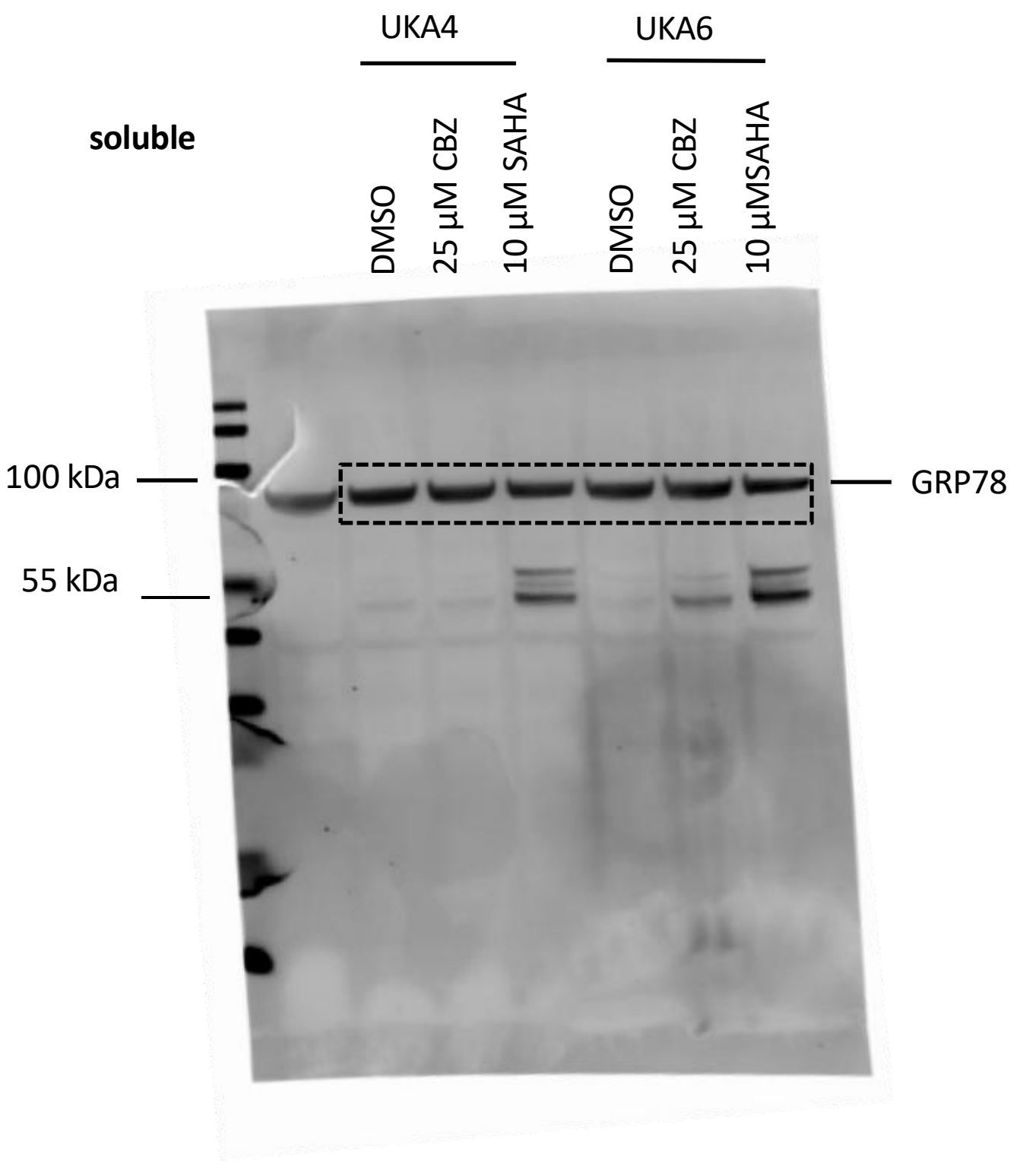

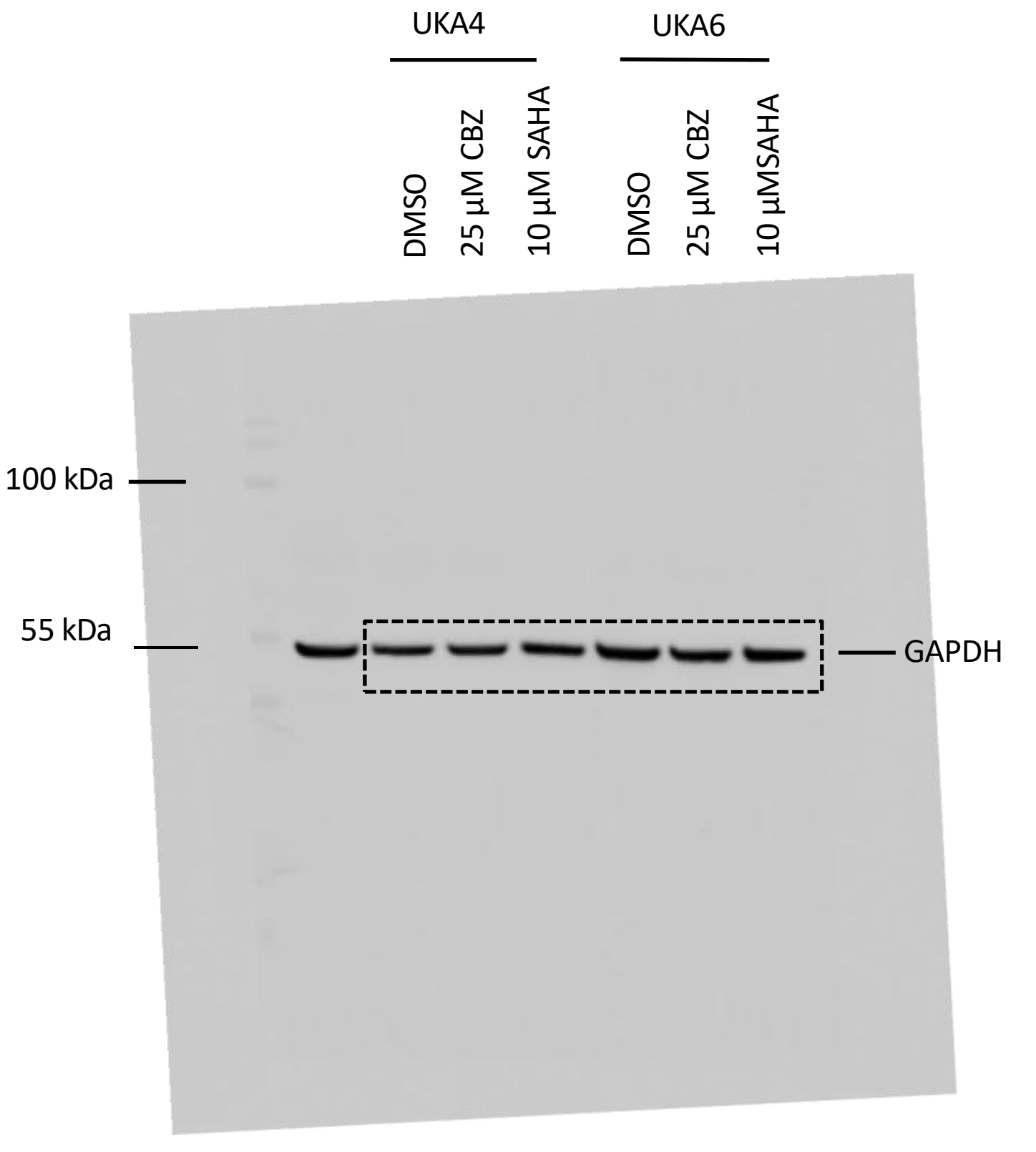

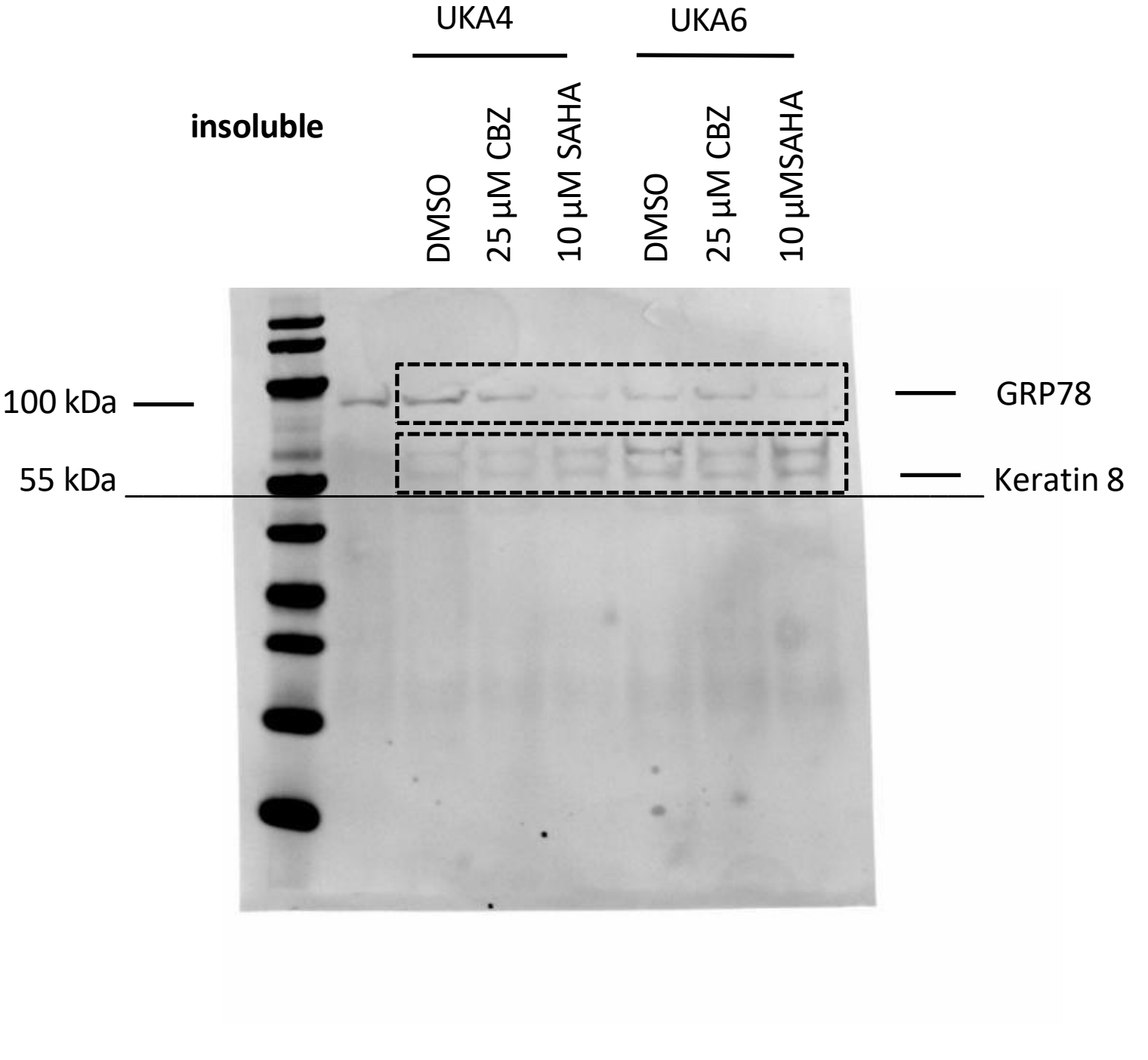

Suppl. Fig. S13: Uncropped Western blots from Suppl. Fig. S10. Dashed lines indicate cropped regions.
