# Supplementary material for: SAHA increases chaperone expression and reduces Z-alpha-1-antitrypsin polymers in a patient specific iPSC-based liver model for alpha-1-antitrypsin deficiency
Source: Stem Cell Res Ther. 2026 Oct 27;17:386. doi: 10.1186/s13287-026-05365-y (PMC13718072; doi:10.1186/s13287-026-05365-y)
Supplement: Supplementary file 2 [file 13287_2026_5365_MOESM2_ESM.pdf]

Supplementary Materials and Methods

Supplementary Table S1: List of primary antibodies used for ICC and WB

Primary Antibody	Species	Dilution ICC	Dilution WB	Company	Catalogue number
**anti-AAT**	goat	1:1,000	1:3,000	GeneTex	GTX27633
**anti-β-Actin**	mouse		1:5,000	Cell Signalling Technology	3700S
**Anti-AFP**	rabbit	1:300		Sigma	HPA023600
**anti-ALB**	mouse	1:100	1:5,000	Sigma	A6684-.2ml
**anti-GAPDH**	rabbit	1:200	1:1,000	Cell Signalling Technology	2118S
**anti-HNF4α**	rabbit	1:250		Abcam	92378
**anti-GRP78**	mouse		1:1,000	BD	610978
**anti-Keratin 8**	mouse		1:1,000	Progen	61038
**anti-γ-Tubulin**	mouse		1:1,000	Sigma	T5326
**Anti SOX17**	goat	1:50		R&D Systems	AF1924
**anti-ZAAT**	mouse	1:50	1:50 – 1:500	HycultBiotech	HYC-HM2289-100UG

Supplementary Table S2: List of secondary antibodies used for ICC and WB

Secondary Antibody	Species	Dilution	Company
**anti-mouse 488**	goat	1:500	Invitrogen
**anti-mouse 555**	goat	1:500	Invitrogen
**anti-rabbit 488**	goat	1:500	Invitrogen
**anti-rabbit 647**	goat	1:500	Invitrogen
**anti-goat 488**	donkey	1:500	Invitrogen
**anti-goat 680RD**	donkey	1:10,000	LI-COR Biosciences
**anti-mouse 680RD**	donkey	1:10,000	LI-COR Biosciences
**Anti-mouse HRP**	goat	1:1000	Thermofisher

Supplementary Table S3: List of primers

Primer		Sequence 5‘→ 3‘	Product size (bp)
ALB	forward	AGCTGTTATGGATGATTTCGCAG	77
	reverse	CCTCGGCAAAGCAGGTCTC	
AFP	forward	AGCAGCTTGGTGGTGGATGA	88
	reverse	CCTGAGCTTGGCACAGATCCT	
AGXT	forward	ACCATCACACAATCCCCGTC	222
	reverse	CTCTCCAGTCATAGCCAGCG	
BAAT	forward	CCTCCTTGGCCTTGGCTTAC	201
	reverse	TGGCTGTGACTTGCTTTAGGT	
CYP3A4	forward	GTGACTTTGCCCATTGTTTAGAAAG	79
	reverse	CAGGCGTGAGCCACTGTG	
CYP3A7	forward	GATTCTGTACGTGCATTGTGCTC	77
	reverse	ATTTGGTCATCTCCTCTATATTACCAAGT	
HAO2	forward	CTGCAAGGGTGAACATGGTG	106
	reverse	TCGATTGATCTCAGCGACCG	
HNF4α	forward	GCACTCGAAGGTCAAGCTA	157
	reverse	GACTCACACACATCTGCGA	
HSPA1A	forward	AGCTGGAGCAGGTGTGTAAC	154
	reverse	CAGCAATCTTGGAAAGGCCC	
HSPA1B	forward	GCAGGTGTGTAACCCCATCA	177
	reverse	GAGTCCCAACAGTCCACCTC	
HSPA2	forward	GACGACATTGACCGGATGGT	154
	reverse	CGCTAATCTTGCCCCTCAGT	
HSPA5	forward	CCGAGGAGGAGGACAAGAAGG	229
	reverse	TCAAAGACCGTGTTCTCGGG	
RPLP0	forward	TCGACAATGGCAGCATCTAC	110
	reverse	ATCCGTCTCCACAGACAAGG	
**SERPINA1**	forward	GGTCACAGAGGAGGCACCC	76
	reverse	AGTCCCTTTCTCGTCGATGGT	

Next generation sequencing (NGS)

The RNA-seq raw data in FASTQ format received from the BMFZ-GTL core facility was aligned against the GRCh38 genome with the HISAT2 (version 2.1.0) alignment software(1). The essentials of this procedure were described in our previous publication(2) with marginal changes in the annotation files and in the HISAT2 command, adapted from the parameter optimizations of Barruzzo et al. (3):

```
hisat2 -p 7 -N 1 -L 20 -i S,1,0.5 -D 25 -R 5 --mp 1,0 --sp 3,0 -x /home/ww/hisatindex/grch38_r109 -U input.fastq.gz -S output.sam
```

Sorting was achieved by the SAMtools software (4) and summarization per gene by the subread (1.6.1) featureCounts software (5) against the annotations from Ensembl Homo_sapiens.GRCh38.109.gtf using parameters –t exon –g gene_id. For filtering expressed genes a threshold of CPM (counts per million) > 1 in at least one sample was applied before limma voom normalization (6, 7) in R/Bioconductor (8).

Supplementary Table S6: Sequencing depths

samplename	desc	group	total_reads	readlength	exomesize	sequencing_depth
**1128-1_S1_R1_001**	UKA6_CBZ1	CBZ	15475233	100	30000000	51,58411
**1128-2_S2_R1_001**	UKA6_CBZ2	CBZ	14893354	100	30000000	49,6445133
**1128-3_S3_R1_001**	UKA6_SAHA1	SAHA	15883352	100	30000000	52,9445067
**1128-4_S4_R1_001**	UKA6_SAHA3	SAHA	20238853	100	30000000	67,4628433
**1128-5_S5_R1_001**	UKA6_DMSO1	DMSO	15771268	100	30000000	52,5708933
**1128-6_S6_R1_001**	UKA6_DMSO2	DMSO	17579531	100	30000000	58,5984367
**1128-7_S7_R1_001**	UKA4_CBZ2	CBZ	18034208	100	30000000	60,1140267
**1128-8_S8_R1_001**	UKA4_CBZ3	CBZ	13287992	100	30000000	44,2933067
**1128-9_S9_R1_001**	UKA4_SAHA2	SAHA	16592516	100	30000000	55,3083867
**1128-10_S10_R1_001**	UKA4_SAHA3	SAHA	15540071	100	30000000	51,8002367
**1128-11_S11_R1_001**	UKA4_DMSO1	DMSO	18021441	100	30000000	60,07147
**1128-12_S12_R1_001**	UKA4_DMSO2	DMSO	18045071	100	30000000	60,1502367
**1128-32_S20_R1_001**	ctrl_DMSO_1	DMSO	18818465	100	30000000	62,7282167
**1128-33_S21_R1_001**	ctrl_DMSO_2	DMSO	13671472	100	30000000	45,5715733
**1128-34_S22_R1_001**	ctrl_DMSO_3	DMSO	19257810	100	30000000	64,1927

Supplementary References

1. Kim D, Langmead B, Salzberg SL. HISAT: a fast spliced aligner with low memory requirements. Nature methods. 2015;12(4):357-60.
2. Martins S, Müller-Schiffmann A, Erichsen L, Bohndorf M, Wruck W, Sleegers K, et al. IPSC-Derived Neuronal Cultures Carrying the Alzheimer's Disease Associated TREM2 R47H Variant Enables the Construction of an Aβ-Induced Gene Regulatory Network. International journal of molecular sciences. 2020;21(12).
3. Baruzzo G, Hayer KE, Kim EJ, Di Camillo B, FitzGerald GA, Grant GR. Simulation-based comprehensive benchmarking of RNA-seq aligners. Nature methods. 2017;14(2):135-9.
4. Li H, Handsaker B, Wysoker A, Fennell T, Ruan J, Homer N, et al. The Sequence Alignment/Map format and SAMtools. Bioinformatics. 2009;25(16):2078-9.
5. Liao Y, Smyth GK, Shi W. featureCounts: an efficient general purpose program for assigning sequence reads to genomic features. Bioinformatics. 2014;30(7):923-30.
6. Law CW, Chen Y, Shi W, Smyth GK. voom: Precision weights unlock linear model analysis tools for RNA-seq read counts. Genome biology. 2014;15(2):R29.
7. Smyth GK. Linear models and empirical bayes methods for assessing differential expression in microarray experiments. Stat Appl Genet Mol Biol. 2004;3:Article3.
8. Gentleman RC, Carey VJ, Bates DM, Bolstad B, Dettling M, Dudoit S, et al. Bioconductor: open software development for computational biology and bioinformatics. Genome biology. 2004;5(10):R80.
